# Single-Stage Surgery for Persistent Cloaca With Vertebral Defects, Anal Atresia, Cardiac Defects, Tracheoesophageal Fistula or Atresia, Renal Anomalies, and Limb Defects (VACTERL) Association: A Case Report on Avoiding Temporary Colostomy

**DOI:** 10.7759/cureus.82487

**Published:** 2025-04-18

**Authors:** Takayuki Masuko, Toshihiro Yanai, Miki Toma

**Affiliations:** 1 Pediatric Urology, Ibaraki Children's Hospital, Ibaraki, JPN; 2 Pediatric Surgery, Ibaraki Children's Hospital, Ibaraki, JPN

**Keywords:** colostomy avoidance, laparoscopic surgery, persistent cloaca, single-stage surgery, urogenital malformation, vacterl association

## Abstract

The standard treatment for persistent cloaca (PC) in patients with vertebral defects, anal atresia, cardiac defects, tracheo-esophageal fistula or atresia, renal anomalies, and limb defects (VACTERL) association typically involves an initial colostomy to decompress the intestine and prevent urinary tract infections. However, patients with VACTERL association often require multiple surgeries, including gastrostomy for esophageal atresia, open-heart surgery, and spinal surgery requiring a prone position, which can complicate subsequent procedures.

Here, we describe two cases of PC with VACTERL association successfully managed without a temporary colostomy until definitive surgery. Instead of a colostomy, a continuous transanal drainage system was used for bowel decompression. Both patients underwent single-stage laparoscopic anorectoplasty and perineal urogenital mobilization without major complications such as enterocolitis or urinary tract infections.

The conventional approach to PC typically requires three laparotomies: colostomy, definitive repair, and colostomy closure. Our experience suggests that, in selected cases, a single-stage surgical approach without a temporary colostomy may offer a feasible alternative in appropriately selected cases.

## Introduction

Patients with VACTERL association often require multiple surgeries to address various congenital anomalies. VACTERL association refers to a non-random combination of congenital anomalies involving the vertebrae, anus, heart, trachea, esophagus, kidneys, and limbs. It is typically diagnosed when three or more of these anomalies are present. Among female patients with persistent cloaca (PC), approximately 67% have a VACTERL association [1]. The conventional approach to PC involves an initial colostomy to prevent urinary tract infections and provide bowel decompression [2,3]. In such patients, the presence of a colostomy may interfere with subsequent procedures, such as gastrostomy for esophageal atresia, open-heart surgery requiring an incision near the colostomy, or spinal surgery requiring a prone position during the postoperative period.

In our previous study, we applied continuous transanal drainage to selected PC patients with a short common channel but without VACTERL association [4]. However, its feasibility in PC patients with VACTERL association, who often require multiple surgeries for associated anomalies, has not been well established. In this study, we examined its clinical applicability in such cases and evaluated the outcomes. Here, we describe two cases of PC with VACTERL association in which preoperative bowel management was successfully achieved using a continuous transanal drainage system. This approach allowed for definitive repair without the need for a temporary colostomy.

## Case presentation

Case 1

A female neonate (birth weight: 2562 g) was delivered at 34 weeks and five days of gestation. On the day of birth, she underwent esophageal anastomosis for type-C tracheoesophageal fistula and duodenal anastomosis for duodenal atresia. The transanal catheter was placed immediately after these procedures during the same anesthesia session. This approach minimized the total anesthesia time and potentially reduced the risk of surgical site infection. The catheter was inserted under general anesthesia with cystoscopic guidance, and contrast fluoroscopy was performed to confirm the anatomy and catheter position. Cystoscopy revealed a persistent cloaca (PC) with a 22-mm common channel and a 20-mm urethra. She was also diagnosed with a ventricular septal defect (VSD) requiring future cardiac surgery and a small right kidney suspected to be a multicystic dysplastic kidney (MCDK). To avoid a temporary colostomy, an 8-Fr open-ended balloon-tipped silicone catheter (Create Medic Co., Ltd., Tokyo, Japan) was inserted into the rectum via the cloacal orifice for continuous bowel decompression. Catheter replacements were performed under fluoroscopic guidance, using a guide wire when necessary. The catheter was secured within a diaper, and rectal irrigation with warm water was performed three times daily at home. Since the patient was able to void spontaneously without signs of a high-pressure bladder or hydrocolpos, no urinary decompression was required. She was managed with standard diaper care. The patient was discharged after caregivers were trained to perform rectal irrigation and manage the drainage system at home. Catheter replacement was conducted every two months under fluoroscopic guidance, with gradual upsizing to 14 Fr. At nine months of age, she underwent untethering surgery for spinal cord abnormalities, which required a prone postoperative position. At 11 months, the drainage catheter was accidentally removed and had to be reinserted under general anesthesia. At 12 months, she successfully underwent laparoscopic anorectoplasty and perineal urogenital mobilization without prior colostomy. She is now over three years old, undergoing potty training, and has no complaints of fecal incontinence.

Case 2

**Figure 1:**
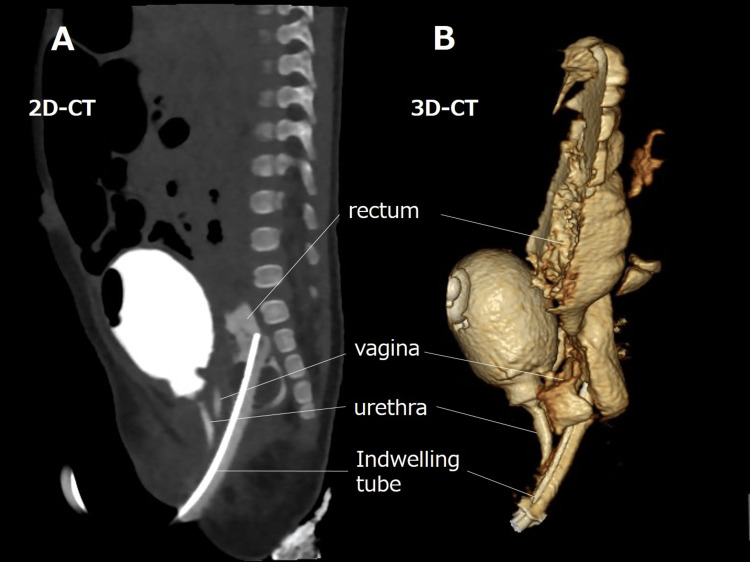
Preoperative imaging of cloacal malformation (A) Sagittal 2D CT cloacagram showing the dilated common channel and the transanally indwelling drainage tube. (B) 3D reconstructed CT cloacagram clearly visualizing the rectum, vagina, and urethra converging into the common channel.

**Figure 2:**
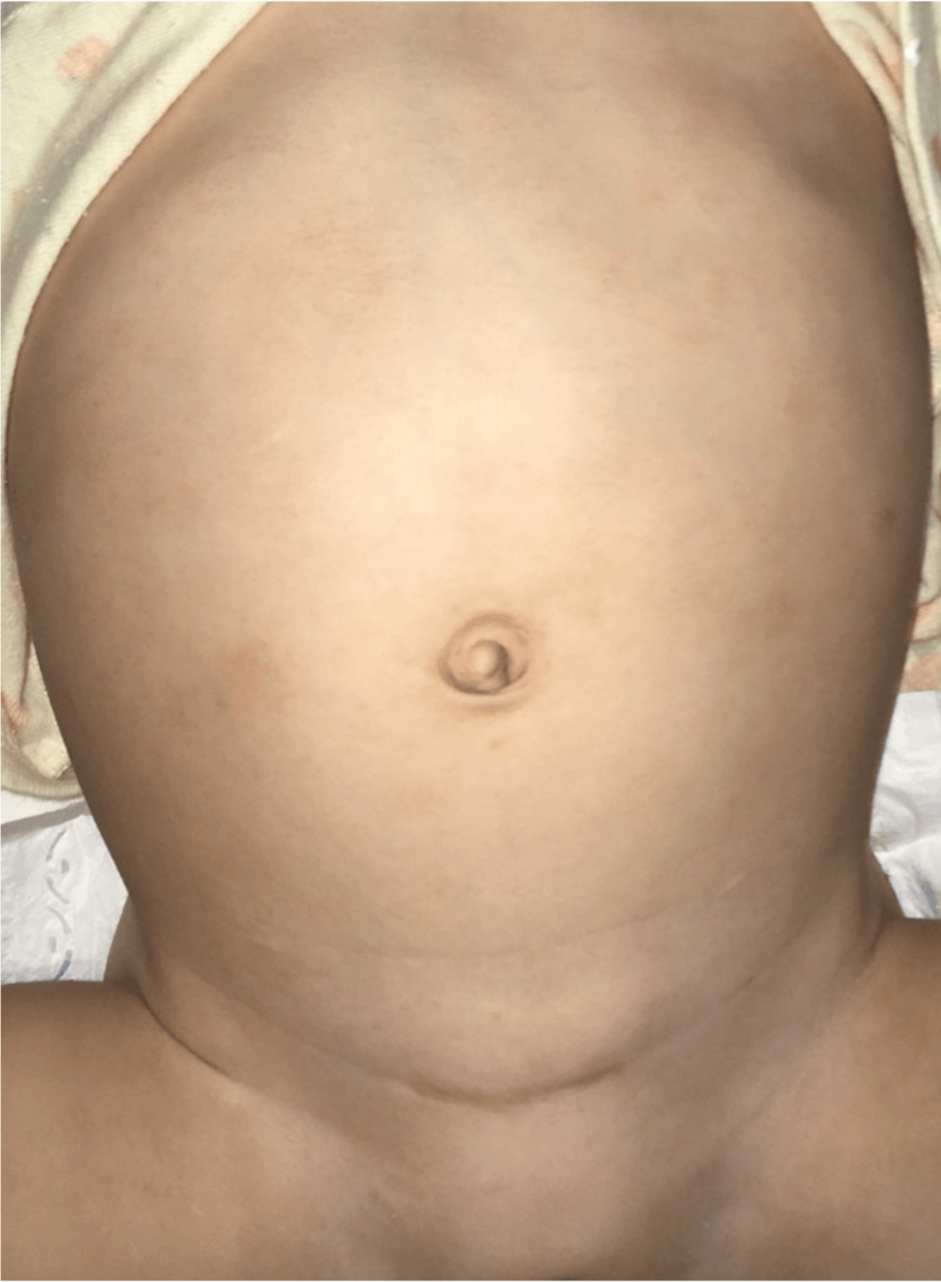
Postoperative abdominal appearance The patient's abdomen in Case 2 at 18 months of age, showing minimal postoperative scarring.

A female neonate (birth weight: 2021 g) was delivered at 37 weeks and one day of gestation. On the day of birth, she underwent esophageal anastomosis for type-C tracheoesophageal fistula. In Case 2, the catheter was similarly placed during the same anesthesia session as the esophageal anastomosis, providing comparable benefits in minimizing operative exposure and reducing infection risk. Cystoscopy revealed a PC with a 12-mm common channel and a 10-mm urethra. She also had severe bilateral vesicoureteral reflux (VUR), requiring future ureteral reimplantation, and spinal abnormalities necessitating untethering surgery. To avoid a temporary colostomy, a 14-Fr open-ended balloon-tipped silicone catheter was inserted transanally for continuous bowel decompression. Rectal irrigation with warm water was performed three times daily at home. The catheter was replaced monthly under fluoroscopic guidance using a guidewire when necessary. A bladder catheter was initially placed but was removed at one month of age as it was no longer required for bladder or vaginal decompression. Continuous antibiotic prophylaxis had been administered. At two months, 3D-CT cloacagraphy was performed for surgical planning (Figure 1). At four months, preoperative measurement revealed a 17-mm urethra, and she successfully underwent laparoscopic anorectoplasty and perineal urogenital mobilization. Prophylactic antibiotics were administered to prevent infections, and no episodes of enterocolitis or urinary tract infections were observed. She subsequently underwent untethering surgery at six months and open ureteral reimplantation at 11 months. Caregivers were satisfied with the postoperative abdominal aesthetics (Figure 2). At 2.5 years of age, she has no evidence of fecal incontinence. However, she has a prolonged voiding interval following postoperative spinal cord untethering, and is managed with clean intermittent catheterization.

## Discussion

Patients with persistent cloaca (PC) and VACTERL association often require multiple surgeries due to associated cardiac, spinal, and urological anomalies. In such cases, the presence of a colostomy can complicate surgical planning and interfere with procedures such as gastrostomy for esophageal atresia, cardiac surgery, or spinal surgery in the prone position. Therefore, single-stage definitive repair without a temporary colostomy may be beneficial in carefully selected patients.

Performing transanal catheter placement during the same anesthesia session as other required surgeries may reduce total anesthesia exposure and lower the risk of surgical site infections. This strategy also facilitates surgical scheduling and minimizes the overall burden of care on both patients and caregivers.

Among patients with anorectal malformations, VACTERL association is observed in approximately 45.6% of cases and cloaca in 25.8% [1]. Cardiac anomalies occur in 40-80% [5], and spinal or urological anomalies frequently require multiple interventions. Avoiding stomas in these patients may reduce the number of abdominal surgeries and help preserve abdominal anatomy for future reconstructions, such as rectal pull-through or vaginal replacement [6]. Additionally, stoma placement can interfere with postoperative positioning in spinal surgeries and is generally avoided by cardiac surgeons in neonates. In both of our cases, the avoidance of colostomy also resulted in improved postoperative abdominal aesthetics, which was appreciated by caregivers.

Anatomical criteria for single-stage repair are still evolving. A common channel length of less than 3 cm and a urethral length of at least 15 mm are considered favorable for total urogenital mobilization, as these features are associated with lower risks of postoperative incontinence [7]. However, precise urethral measurement in neonates is challenging, and values may vary depending on timing and technique. In our approach, we used initial thresholds of a common channel <3 cm and urethral length ≥10 mm as provisional criteria. We anticipated anatomical growth and conducted a re-evaluation prior to definitive surgery. If the urethra appeared clearly short during reassessment, the surgical plan was modified accordingly by the attending physician. Given that this report includes only our second experience with this approach, these criteria should be considered preliminary and subject to refinement through further clinical experience.

Continuous colonic decompression using an indwelling transanal catheter is a well-established preoperative management strategy in conditions such as Hirschsprung disease, with no evidence of adverse effects on long-term bowel function [8,9]. Similarly, in our cases, we observed no signs of impaired bowel function following catheter management. Although longer follow-up is needed, early outcomes are encouraging. Complications such as enterocolitis and vaginitis were not observed during the management period in either case. In the neonatal period, transanal catheter management using a double diaper system was feasible, and the burden of rectal irrigation was comparable to that of stoma care. Unlike the conventional three-stage approach (colostomy, definitive repair, and stoma closure), our strategy potentially reduces the number of surgical interventions.

Despite its advantages, this approach has limitations. One major concern is the risk of accidental catheter dislodgement, which may require reinsertion under general anesthesia, as seen in Case 1. Preoperative imaging can also be challenging, particularly in obtaining sufficient rectal distension for cloacagram studies [6]. In addition, the catheter replacement intervals varied between the two cases, reflecting individual physician preferences and adaptations based on prior experience. As this method is still evolving, standardized protocols have not yet been established. Furthermore, longer follow-up is required to assess functional outcomes, such as fecal continence and urinary tract function. Given the small sample size, criteria for candidate selection, balloon size, and catheter management remain provisional. Further studies with larger patient cohorts are needed to evaluate the safety, efficacy, and practical guidelines for this novel strategy.

## Conclusions

Single-stage definitive surgery without a temporary colostomy is feasible in selected cases of PC with VACTERL association using transanal catheter placement for bowel decompression. This approach may reduce surgical burden and improve cosmetic outcomes. While challenges such as catheter dislodgement remain, our findings support further investigation in larger cohorts with long-term follow-up.
